# Stimulus-induced dissociation of neuronal firing rates and local field potential gamma power and its relationship to the blood oxygen level-dependent signal in macaque primary visual cortex

**DOI:** 10.1111/j.1460-9568.2011.07877.x

**Published:** 2011-12

**Authors:** M J Bartolo, M A Gieselmann, V Vuksanovic, D Hunter, L Sun, X Chen, L S Delicato, A Thiele

**Affiliations:** 1 Institute of Neuroscience, Newcastle UniversityNewcastle upon Tyne, NE2 4HH, UK; 2 Department of Psychology, School of Business, Law & Psychology, University of SunderlandSunderland, UK

**Keywords:** BOLD, cross-orientation inhibition, fMRI, LFP, spiking, V1

## Abstract

The functional magnetic resonance imaging (fMRI) blood oxygenation level-dependent (BOLD) signal is regularly used to assign neuronal activity to cognitive function. Recent analyses have shown that the local field potential (LFP) gamma power is a better predictor of the fMRI BOLD signal than spiking activity. However, LFP gamma power and spiking activity are usually correlated, clouding the analysis of the neural basis of the BOLD signal. We show that changes in LFP gamma power and spiking activity in the primary visual cortex (V1) of the awake primate can be dissociated by using grating and plaid pattern stimuli, which differentially engage surround suppression and cross-orientation inhibition/facilitation within and between cortical columns. Grating presentation yielded substantial V1 LFP gamma frequency oscillations and significant multi-unit activity. Plaid pattern presentation significantly reduced the LFP gamma power while increasing population multi-unit activity. The fMRI BOLD activity followed the LFP gamma power changes, not the multi-unit activity. Inference of neuronal activity from the fMRI BOLD signal thus requires detailed *a priori* knowledge of how different stimuli or tasks activate the cortical network.

## Introduction

Functional magnetic resonance imaging (fMRI), using the blood oxygenation level-dependent (BOLD) signal, is ubiquitously used to study brain function, and inferences are often made regarding the underlying changes in neuronal activity (Boynton *et al.*, 1996; Heeger *et al.*, 2000; Logothetis *et al.*, 2001; Logothetis, 2002, 2003; Mukamel *et al.*, 2005). Early work argued in favour of a linear transform model, whereby the fMRI BOLD response is a time-averaged linear transform of neuronal (spiking) activity within a given brain volume (Boynton *et al.*, 1996; Rees *et al.*, 2000). Other studies have argued that spiking activity and blood flow changes are best described by non-linear transformation (Devor *et al.*, 2003, 2005), without challenging the basic assumption that increases in fMRI signals reflect increased neuronal spiking activity. More recently, a variety of fMRI and optical imaging studies have argued that the fMRI BOLD signal is better correlated with changes in the local field potential (LFP), which reflect synchronized local changes in subthreshold synaptic activity (dendrosomatic currents) (Mitzdorf, 1987), than with the spiking activity (axo-somatic currents) of neurons (Logothetis *et al.*, 2001; Logothetis, 2002, 2003; Kayser *et al.*, 2004; Niessing *et al.*, 2005; Wilke *et al.*, 2006; Viswanathan & Freeman, 2007; Lippert *et al*., 2010). The correlation between local blood oxygenation level changes and the LFP signal is particularly prominent when the LFP gamma frequency range (∼25–80 Hz, gLFP; although note we restrict our analysis to the frequency band of 30–60 Hz) is used for analysis (Logothetis *et al.*, 2001; Niessing *et al.*, 2005; Viswanathan & Freeman, 2007; Goense & Logothetis, 2008; Lippert *et al*., 2010). Experimentally induced dissociations between firing rate and gLFP and the associated changes in fMRI BOLD signal or regional cerebral blood flow have corroborated the view that changes in regional cerebral blood flow or BOLD are strongly linked to gLFP, not to the local firing rate (Mathiesen *et al.*, 2000; Rauch *et al.*, 2008). It could be argued that these studies have little impact on the interpretation of the BOLD signal in fMRI studies, as stimulus-induced changes in spiking and gLFP often co-vary (Logothetis *et al.*, 2001; Logothetis, 2002, 2003; Henrie & Shapley, 2005; Liu & Newsome, 2006). However, recent studies have reported stimulus-induced or task-induced de-correlations or anti-correlations of gLFP and firing rates (Logothetis *et al.*, 2001; Viswanathan & Freeman, 2007; Gieselmann & Thiele, 2008; Lima *et al.*, 2009; Chalk *et al.*, 2010; Lippert *et al*., 2010). An anti-correlation between gLFP and firing rate changes is predictable for a variety of stimulus and task conditions, as gLFP oscillations are assumed to be determined by the summed contributions of excitatory and inhibitory activity from both the classic and extra-classic receptive field (RF), with recurrent inhibitory activity playing a dominant role (Traub *et al.*, 1996; Whittington & Traub, 2003; Tiesinga & Sejnowski, 2004; Gieselmann & Thiele, 2008). Thus, strong recurrent inhibition drives gLFP oscillations, while at the same time possibly limiting spiking activity (Gieselmann & Thiele, 2008).

The latter can be induced in the primary visual cortex (V1) by presenting a single grating that engages the centre–surround inhibition (Gieselmann & Thiele, 2008). Addition of a second orthogonal grating results in a plaid pattern. Plaid pattern presentation could invoke cross-orientation inhibition (Gilbert & Wiesel, 1990; Cavanaugh *et al.*, 2002a; Jones *et al.*, 2002), but also cross-orientation facilitation (Gilbert & Wiesel, 1990; Cavanaugh *et al.*, 2002b; Jones *et al.*, 2002). Presenting the additional grating will affect neurons that were stimulated more or less optimally by the single grating, but also neurons that were stimulated sub-optimally by the single grating. The close to optimally stimulated group might exhibit a slight reduction or no change in overall firing rate when the plaid pattern is presented, owing to cross-orientation inhibition. However, the non-optimally stimulated group should increase their overall firing rates upon plaid pattern presentation, as the additional grating will be closer to their preferred orientation, thereby increasing excitatory drive. If averaged across a V1 hypercolumn, plaid pattern presentation should result in overall higher spiking activity. Conversely, gLFP in V1 has been shown to be strongly reduced upon plaid pattern stimulus presentation (Lima *et al.*, 2009). Thus, we predict a profound stimulus-induced dissociation between gLFP and multi-unit activity when comparing single-grating-induced vs. plaid pattern-induced activity in V1.

Here, we exploited this predicted dissociation to investigate the neuronal basis of the fMRI BOLD signal in V1 of the awake macaque monkey. Our data show that fMRI BOLD signal changes correlated with gLFP in V1, not with neuronal spiking activity, when a simple stimulus manipulation from single-grating to plaid pattern presentation was made. The simple assumption that decreases or increases in fMRI BOLD activity are associated with decreases or increases in neuronal activity can be misleading. Thus, a detailed understanding of stimulus-induced (or task-induced) changes of neuronal activity is necessary for adequate interpretation of fMRI BOLD data.

## Materials and methods

All experiments were carried out in accordance with the European Communities Council Directive 1986 (86/609/EEC), the US National Institutes of Health Guidelines for the Care and Use of Animals for Experimental Procedures, and the UK Animals Scientific Procedures Act. Two animals (monkeys C and W) were used for the fMRI experiments, and three animals (monkeys F, D, and B) were used for the electrophysiology experiments.

### Surgical preparation

Monkeys (*Macaca mulatta*, male, 5–11 years old) were implanted with a head holder. Animals used in electrophysiological experiments were additionally implanted with an eye coil, and recording chambers above V1 (or a Blackrock microelectrode array in V1). All surgical procedures were performed under general anaesthesia and in sterile conditions. All details regarding surgical procedures, postoperative care and the cleaning of the implant and recording chambers have been published elsewhere (Thiele *et al.*, 2006).

### Electrophysiological recordings

We used tungsten-in-glass microelectrodes (0.5–2 MΩ, made in-house) for recording of extracellular spiking activity and LFP in monkeys F and D. In monkey B, we recorded neuronal activity in V1 from a chronically implanted 4 × 5 Cereport microelectrode array (Blackrock Microsystems, Salt Lake City, Utah, USA). Remote Cortex 5.95 (http://www.cortex.salk.edu) was used for stimulus presentation and behavioural data collection. Neuronal data were collected with Cheetah data acquisition software (Neuralynx, Bozeman, MT, USA) interlinked with Remote Cortex 5.95. Spike waveforms were sampled at 30 kHz. In post-processing, spike times were sampled at 1-kHz resolution. LFP data were bandpass filtered (1–475 Hz) and sampled continuously at a sampling rate of 1.017 kHz. Spiking and LFP data were extracted from the ‘steady-state’ spontaneous period (−512 to 0 ms before stimulus onset) and the steady-state response period (200–3000 ms following stimulus onset).

### Behavioural details

#### fMRI

Monkeys were trained to keep fixation (eye window, 5° diameter) while either a horizontally oriented sinusoidal grating or a plaid pattern stimulus was presented at 6.5° to the right of the vertical meridian. Eye position was monitored with a camera-based system (SMI, 60-Hz sampling rate; SensoMotoric Instruments, Teltow, Germany). Following fixation onset, stimuli were presented for 15 s, and this was followed by an 18-s blank period (33 s in total per trial). While animals fixated throughout the trial, they received a reward every 3 s, and they received an additional barrage of rewards at the end for adequate performance. Only trials with adequate fixation performance were analysed further.

#### Electrophysiology

Monkeys were trained to keep fixation (eye window, 2° diameter) while either a horizontally oriented Gabor or a plaid pattern stimulus was presented superimposed on the RF of V1 neurons. Eye position was monitored with a camera-based system (Thomas Recording, 220-Hz sampling rate; Europaviertel, Giessen, Germany) with a fixation window of ± 0.5–0.7° in monkeys D and B, and with a scleral search coil in monkey F (250-Hz sampling rate). After presentation of a fixation spot, trials contained an initial 500-ms blank period, before the stimulus was presented for 3000 ms.

### Visual stimuli and presentation

We presented either a moving single Gabor grating, or a plaid pattern composed of horizontal and vertical counter-phase reversing Gabor gratings. Stimuli had an overall diameter of 3°. Each grating had a 0.5 cycle/degree spatial frequency, 4-Hz temporal frequency, and a *σ* of 0.5 (Gaussian envelope widths). During the fMRI experiments, stimuli were centred at 6.5° eccentricity along the horizontal meridian in the right visual field. In the electrophysiological experiments, stimuli of size and composition identical to those used in the fMRI experiments were centred at the RF of the recorded neurons in monkeys D and F. In monkey B, the stimulus was centred on the aggregate RF from the 15 recording sites (14 sites for the recordings where stimulus orientation was parametrically varied; see below). Given that the electrode grid was 4 × 4 electrodes with an interelectrode spacing of 300 μm, the RFs were very close together and had substantial overlap. The average stimulus eccentricity was 4.71° in monkey F, 4.23° in monkey D, and 4.61° in monkey B.

#### fMRI

An experimental session (run) consisted of 20 trials of grating and plaid pattern presentation each, and lasted for ∼22 min. Stimuli were presented alternately, and consisted of 15 s of stimulus presentation and 18 s with no stimulus. Stimuli were presented and synchronized with data recording by the use of VCortex 2.2 (http://www.cortex.salk.edu). For the initial recordings, we used an AVOTEC (Avotec, Stuart, FL, USA) projection system, which provided a field of view (FOV) of 31.3 × 24.2°. Owing to the image quality in terms of contrast and colour homogeneity, and difficulties in adequate contrast and size calibration, we changed to a back projector-based system soon after. By use of the back projector system, the image was projected onto a screen above the monkey's head with a liquid crystal display projector (NEC NP 1150, 1024 × 768 visual resolution, 60-Hz refresh rate). The monkey viewed the projected visual image through a mirror mounted in front of his eyes. The whole-screen image had 19.45 × 14.65 of the visual angle. The stimulus (Michelson) contrast was 96%, 48%, 24%, 16%, 12%, or 8%. The individual gratings in the plaid pattern were generated such that the maximum contrast of the plaid pattern reached the contrast values of the single-grating.

#### Electrophysiology

Monkeys fixated a red fixation point (0.1° in diameter) on a grey background (21 cd/m^2^) presented centrally on a 20-inch analogue cathode ray tube monitor (85 or 110 Hz, and 1600 × 1200 or 1280 × 1024 pixels, 57 cm from the animal). The stimulus (Michelson) contrasts were 96%, 48%, 32%, 24%, 16%, 12%, 8%, and 4%. In a separate set of experiments, we presented stimuli at three different contrasts (16%, 32%, and 64%) and with nine different grating orientations (0°, 20°, 40°, 60°, 80°, 100°, 120°, 140°, and 160°; the same orientations with a 90° rotated and superimposed orientation were used for the plaid pattern). The orientation tuning of neurons was defined as the response to the orientation that was furthest (80°) from the orientation that yielded the largest response (preferred orientation) divided by the response to the preferred orientation after subtraction of spontaneous activity.

### RF and neuronal preference characterization

For each recording site, we initially determined the location of the RF as well as the optimal orientation, spatial frequency and phase, using reverse correlation techniques (DeAngelis *et al.*, 1994; Gieselmann & Thiele, 2008). Briefly, the location of the RF was estimated by mapping the classic RF with briefly presented dark and light squares (0.1° in width, 100% contrast) at pseudorandom locations on a 10 × 10 grid (a 1° × 1° area). The RF centre was taken as the location of the peak as estimated from the spatial activity maps displayed online. Thereafter, the tuning properties were estimated with the use of static sinusoidal gratings (1° in diameter) centred on the RF. These gratings varied in orientation (12 orientations, 0–165°), spatial frequency (1, 3, 5, 7, 8, 9 or 10 cycles/degree) and phase (0, 0.5π, π and 1.5π) every 60 ms in a pseudorandomized order. The stimulus that yielded the peak response was taken to represent the preferred orientation, spatial frequency and phase of the neuron under study. These reverse correlation procedures were conducted while monkeys fixated centrally on the cathode ray tube monitor.

### Electrophysiological data analysis

#### Spiking analysis

Spontaneous spiking activity was analysed in a time window from −512 ms before stimulus onset until stimulus onset. Stimulus-driven spiking activity was analysed in a time window from 200 ms until 3000 ms after stimulus onset. Normalization of spiking activity was performed as follows. First the mean spontaneous activity (spikes/s) was subtracted from the mean stimulus-driven activity (spikes/s). Then, the (mean) activity given a specific stimulus was divided by the maximum stimulus-driven activity (i.e. the activity that the ‘optimal’ stimulus elicited). Non-normalized spiking activity was calculated as the mean activity (spikes/s) averaged over the analysis period (200–3000 ms after stimulus onset) given a specific stimulus.

#### LFP analysis

For each trial, the power spectrum of the LFP response over the time period −512 to 0 ms relative to stimulus onset [yielding the baseline power spectrum (BPS)] and the period 200–3000 ms after stimulus onset (yielding the stimulus power spectrum) was estimated with multi-taper techniques (Percival & Walden, 1993) [time–bandwidth product, 3; tapers, 5; Chronux toolbox (http://chronux.org/)] or with a fast Fourier transform (with Hanning window). The mean and standard deviation of the BPS (BPS_M_ and BPS_SD_) were obtained from all of the power spectra of the baseline window from all trials. The mean stimulus power spectrum (SPS_M_) for a specific stimulus was obtained by averaging the single trial power spectra obtained from that stimulus presentation. The stimulus-induced power spectrum (PS_*z*_) was expressed as the *z*-score of BPS:


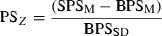
(1)

To analyse changes in power in specific frequency bands, we subdivided the spectra into the alpha (7–13 Hz), beta (13–25 Hz) and gamma (30–60 Hz) frequency bands. We averaged the power within these different bands for each recording site, and calculated the population power spectra by averaging across the different sites.

The influence of stimulus contrast and stimulus type (grating vs. plaid) on firing rates and on LFP band-limited power spectra was determined by using a two-factor anova (for experiments where a fixed stimulus of different contrasts was presented). We performed the anova on normalized and on non-normalized spiking data. A three-factor anova was used for the experiments where we varied stimulus type (factor 1, grating vs. plaid), stimulus orientation (factor 2), and contrast (factor 3).

### Magnetic resonance imaging (MRI) hardware and imaging

Data were recorded with an actively shielded and vertical scanner (4.7 T, Bruker Biospec 47/60 VAS, inner-bore width of 38 cm) that was equipped with a Bruker GA-385 gradient system (Bruker Medical, Ettlingen, Germany). To obtain a better signal/noise ratio, a custom-made transceiver surface coil was placed over the left occipital hemisphere. Alternatively, we used a Bruker receiver surface coil. For monkey C, all experiments with 48% contrast stimuli and half of the experiments with 96% contrast stimuli were collected with the transceiver surface coil. For monkey W, all data related to the lower contrasts stimuli (8–24% contrast) and three runs of the 48% contrast stimulus were acquired with the receiver coil. This change in surface coils did not affect our main results, and had relatively little effect on the region of interest (ROI) location (see Supporting Information Fig. S1 and associated text for possible reasons for small differences). The percentage BOLD signal change was similar between coils.

MRI scans covered V1 with 12 axial slices (slice thickness, 2 mm) that were placed over the occipital lobe. The exact position was chosen with the help of a sagittal modified driven equilibrium Fourier transform (MDEFT).

Most functional data acquisitions were achieved with single-shot gradient-recalled echo-planar imaging (EPI) sequences, with the following parameters: for monkey W, in-plane resolution of 1 × 1 mm, FOV = 9.6 × 9.6 mm, 12 slices (2 mm), echo time (TE) = 21 ms, and flip angle = 45°; and for monkey C, in-plane resolution of 1 × 2 mm, FOV = 128 × 128 mm, 16 slices (2 mm), TE = 20.9 ms, and flip angle = 20.8°. The different parameters used did not result in any qualitative differences or noticeable quantitative differences. Functional volume acquisition was separated by 3 s [repetition time (TR)]. A block design paradigm was used to detect BOLD activity that was related to the stimulus presentation.

For each session, we collected structural images (T1-weighted) with the same geometry as the functional scans in order to facilitate registration between them. MDEFT sequence image parameters were as follows: TE = 6 ms, TR = 1887.5 ms, flip angle = 30°, and FOV = 128 × 80 mm^2^, with an in-plane resolution of 0.5 × 0.312 mm^2^.

### fMRI data analyses and identification of activated voxels

We discarded any trial where fixation throughout the trial was not accurate. Correct trials were analysed for significant signal changes with fsl (http://www.fmrib.ox.ac.uk/fsl/). To minimize head motions, motion correction (Jenkinson *et al.*, 2002) was implemented, with the middle volume as an initial template image for registration. Head motion parameters were not used as an exclusion criterion for bad trials. Spatial filtering was performed with a Gaussian convolution of 3 mm (full-width half-maximum). We applied high pass temporal filtering by fitting a Gaussian weighted least squares straight line with a sigma = 25.0 s to the data, and low pass filtering by fitting a Gaussian with a half-width at half maximum of 2.8 s to each voxel in the time series. To localize activated voxels, a general linear model analysis was performed, convolving the model with a gamma function [mean lag of 5.6 s and a standard deviation (half-width of the gamma smoothing) of 2.8 s]. Significantly activated voxels (cluster-corrected *z* > 3, *P* < 0.05) that were common in both conditions and localized in V1 were selected for further analysis.

### BOLD time course

**Fig. 5:**
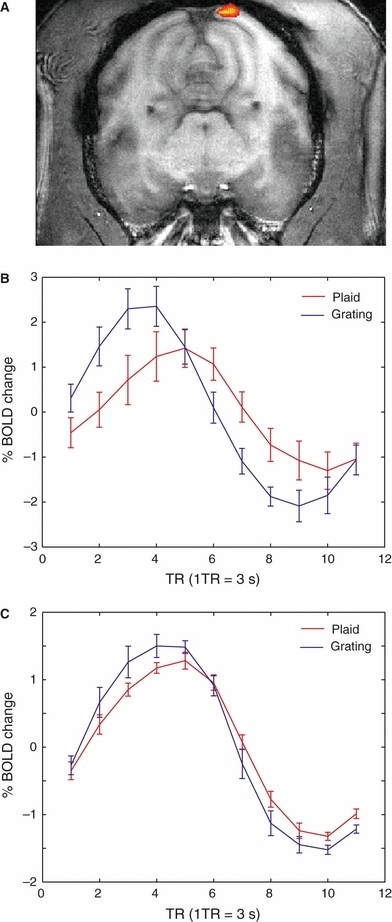
fMRI BOLD signals in V1 of monkey W. (A) Significantly activated voxel in V1 co-registered with a structural MRI scan. (B) Time course of fMRI BOLD signal change from one example session for grating (blue) and plaid pattern (red) presentation. The stimulus contrast was 96%. In this example, a significant interaction was found between stimulus type and time (*F*_10,90_ = 4.665, *P* < 0.001, two-way repeated measures anova). (C) Time course of fMRI BOLD signal change from all sessions where a 96% contrast stimulus was used (*n* = 8). Overall, the percentage BOLD signal change was significantly higher for grating than for plaid pattern presentation (*t*_7_ = −2.552, *P* < 0.05, paired *t*-test). Blue: grating. Red: plaid pattern. Error bars denote SEMs.

**Fig. 6:**
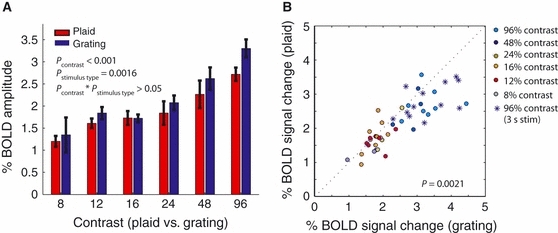
fMRI BOLD signals in V1 of monkey W for different stimulus contrasts and the two types of stimulus. (A) Average fMRI BOLD signal change for different contrasts and stimuli. Contrast and stimulus type significantly affected the BOLD signal change, but there was no interaction between the two factors (mixed anova, *P*-values provided in inset). Error bars denote SEMs. (B) Comparison of fMRI BOLD signal change for all of the experimental sessions and the different contrasts (color coded). The signal change was significantly lower for plaid pattern than for grating presentation. Filled circles denote percentage signal change where the stimulus duration was 15 s; stars denote percentage signal change where the stimulus duration was 3 s.

For each experimental session, we extracted the signal from those voxels that were commonly activated in the grating condition and in the plaid condition (*z* > 3, *P* < 0.05). These aggregate voxels constituted our ROI. For this ROI, we then calculated the percentage BOLD signal change for each run. This was performed using the following calculation:


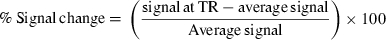


where ‘signal at TR’ corresponds to the EPI intensity obtained in the ROI at a given TR, and ‘average signal’ corresponds to the mean EPI intensity obtained at the ROI across all TRs of that run. Figures 5 and 6 were generated with the criterion described above, where percentage BOLD amplitude corresponds to the mean difference between the maximum and minimum signal change over a run. Other approaches to select our ROI were used (see below), and yielded basically identical results.

Statistical analyses were performed on these BOLD percentage signal change results using spss (IBM) and Matlab (Mathworks), whereby each experimental session contributed two percentage signal change values (one for gratings and one for plaid pattern), given a stimulus contrast. We performed a two-factor anova with these percentage signal change values from all of the experimental sessions (factor 1, stimulus type; factor 2, contrast) to determine whether there was a main effect of stimulus, a main effect of contrast, and an interaction.

For the 96% contrast condition, we also performed a high-level analysis in monkey W (analysis across sessions, *n* = 8). Initially, we generated a mean structural image from the anatomical scans (MDEFT) of each run. This mean structural image was used as the standard space. We then registered the EPI data from all sessions to this standard space, which yielded an average BOLD response across sessions, and allowed for a multi-session analysis of those voxels that were significantly activated by the grating and the plaid pattern. From these voxels, we extracted a common ROI, by using all of the voxels with a *z*-score > 8 under both stimulus conditions (*P* < 0.001). This ROI was then used for each run individually to extract fMRI BOLD signal change. Thus, we ensured that we always used the same number of voxels and used the same region in this analysis. This method gave similar results to the one described above.

We also used a less stringent criterion for our ROI extraction, by using all of the activated voxels of all runs where the *z*-score exceeded 3 (*P* < 0.05) within a given experimental session. We then compiled these voxels into a ‘super ROI’, which was substantially larger than the ROIs of the previous analysis, and was likely to include more noise, but ensured that we did not miss activation from the fringes, which could in principle affect the overall results. However, the difference between the grating and plaid pattern responses in the BOLD signal, as described in Results, was equally present in this approach.

## Results

We first report the results of electrophysiological recordings, and then the results of fMRI experiments.

### Electrophysiology

**Fig. 1:**
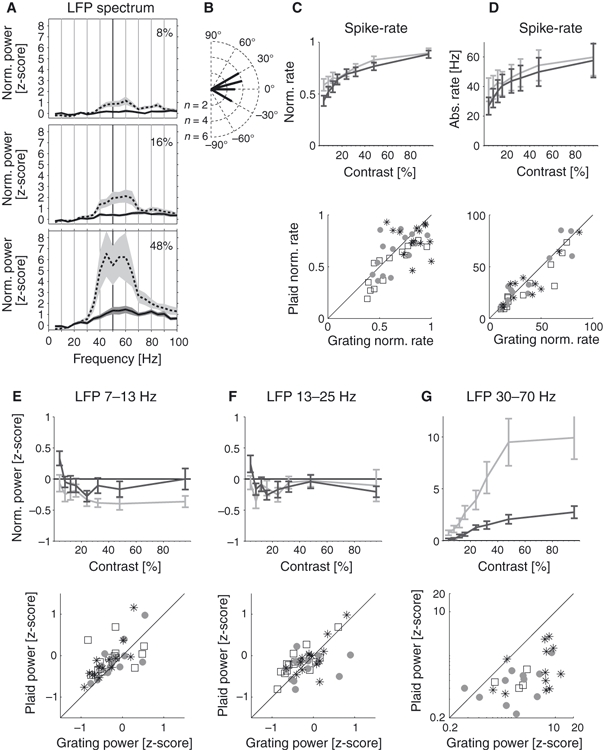
Comparison of LFP power and multi-unit spiking activity for sites with a preferred orientation that was close to the orientation of the grating stimulus. (A) Stimulus-induced LFP power for gratings (dashed line) and plaid pattern (solid line) for different stimulus contrasts (top to bottom). The shaded area represents the standard error of the mean (SEM). (B) Distribution of preferred orientations of this neuronal sample relative to the (horizontal) grating (indicated as 0°). (C) Average normalized spiking activities for different contrasts when gratings (grey) and plaid pattern (black) were presented. Scatter plots of firing rates from individual experiments for three different contrast levels (stars, 48%; grey dots, 24%; open squares, 8% contrast) for the two stimulus types are shown in the bottom subplot. (D) Average non-normalized spiking activities for different contrasts when gratings (grey) and plaid pattern (black) were presented. Scatter plots of firing rates from individual experiments for three different contrast levels (stars, 48%; grey dots, 24%; open squares, 8% contrast) for the two stimulus types are shown in the bottom subplot. (E) Average LFP alpha power for the two stimuli as a function of stimulus contrast (black, plaid pattern; grey, gratings). Scatter plots of LFP alpha power from individual experiments for three different contrast levels (stars, 48%; grey dots, 24%; open squares, 8% contrast) for the two stimulus types are shown in the lower subplot. (F) Same as E, but the data shown are for the beta band. (G) Same as E, but the data shown are for the gamma band. Error bars denote SEMs.

**Fig. 2:**
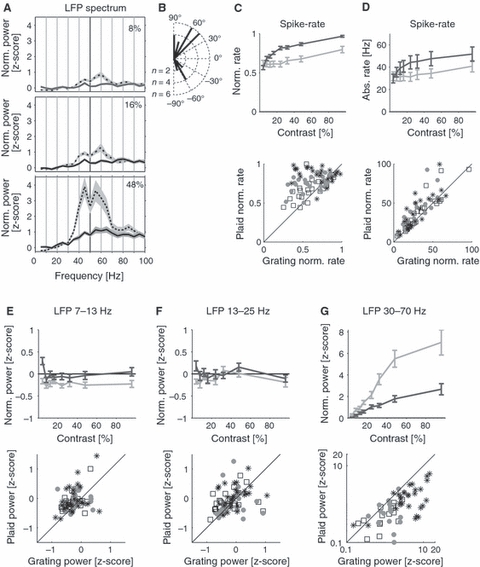
Comparison of LFP power and multi-unit spiking activity for sites stimulated at a non-preferred orientation with the single grating stimulus. (A) Stimulus-induced LFP spectral power for gratings (dashed line) and plaid pattern (solid line) for different stimulus contrasts (top to bottom). The shaded area represents the standard error of the mean (SEM). (B) Distribution of preferred orientations of this neuronal sample relative to the horizontal grating stimulus (indicated as 0°). (C) Average normalized spiking activity for different contrasts when gratings (grey) and plaid pattern (black) were presented. Scatter plots of firing rates from individual experiments for three different contrast levels (stars, 48%; grey dots, 24%; open squares, 8% contrast) for the two stimulus types are shown in the bottom subplot. (D) Average non-normalized spiking activity for different contrasts when gratings (grey) and plaid pattern (black) were presented. Scatter plots of firing rates from individual experiments for three different contrast levels (stars, 48%; grey dots, 24%; open squares, 8% contrast) for the two stimulus types are shown in the bottom subplot. (E) Average LFP alpha power for the two stimuli as a function of stimulus contrast (black, plaid pattern; grey, gratings). Scatter plots of LFP alpha power from individual experiments for three different contrast levels (stars, 48%; grey dots, 24%; open squares, 8% contrast) for the two stimulus types are shown in the lower subplot. (F) Same as E, but the data shown are for the beta band. (G) Same as E, but the data shown are for the gamma band. Error bars denote SEMs.

We recorded multi-unit activity and LFP from a total of 49 recording sites in three monkeys (monkey F, 22; monkey D, 12; monkey B, 15). In most of the experiments, visual stimuli were not optimized to the orientation preference of the neuronal sites recorded. The reasoning behind the non-optimization approach is that it approximates conditions in the fMRI experiment, where a stimulus of fixed orientation would activate neurons within a voxel that have substantial variation in their orientation preference. However, we determined the preferred orientation of each site by using a reverse correlation technique (see Materials and methods). This allowed us to analyse neuronal firing rate and gLFP for sites that were stimulated more or less optimally by the single grating, that is, when the grating orientation was close to the preferred orientation of the site (± −30° from the site's preferred orientation, *n* = 14), as well as to perform the analysis for sites where the grating orientation was > 30° away from the site's preferred orientation (*n* = 27). The pool of neurons that was stimulated close to their preferred orientation (pool 1 in Table 1) might show signs of cross-orientation inhibition when the plaid pattern was presented, and this might somewhat decrease their overall firing rate (although cross-orientation facilitation could also occur). Conversely, the pool of neurons that was stimulated with an orientation that differed from their preferred orientation (pool 2 in Table 1) should increase their firing rate upon plaid pattern presentation, as they would now confronted with a grating that contained a more preferred component. Figure 1A illustrates the averaged stimulus-induced LFP spectrum for the sites that were stimulated optimally or non-optimally with the single grating (pool 1) for three different stimulus contrasts. As stimulus contrast increased, the stimulus-induced spectra showed an increase in the gamma range, particularly for single grating stimuli. Plaid pattern also showed an increase, but it appeared to be less profound. Figure 1B shows the distribution of preferred orientation relative to the grating orientation (which was horizontal; labelled as 0° in Fig. 1B). Figure 1C shows the average normalized firing rate for the two stimulus conditions and the different contrasts. Figure 1D shows the average non-normalized firing rate for the two stimulus conditions and the different contrasts. As expected, firing rates increased with increasing contrast (two-factor anova, *P* < 0.001, contrast main effect). Firing rates were slightly higher for the single grating presentation than for the plaid pattern presentation, indicative of cross-orientation inhibition. The difference was relatively small, but it was significant (see Table 1 for the significance of stimulus main effect for normalized and non-normalized rates). A breakdown of these effects for the different recording sites is shown in Fig. 1C and D (bottom) for three contrast level (8%, 16%, and 48%). For the data shown, the addition of a second orthogonal grating to a single grating more often reduced firing rates than increased it, as compared with the single-grating response. A signed rank test revealed that this was significant (*P* < 0.05) for the normalized and non-normalized data. Both stimulus types caused a significant reduction in the band-limited power in the alpha band (Fig. 1E; Table 1). The alpha band power reduction depended significantly on contrast (*P* < 0.001; Table 1; higher contrast reduced the alpha band power). The beta band (Fig. 1F) was unaffected by stimulus type and contrast (see Table 1 for details of significance). The largest stimulus-induced changes appeared to occur in the gamma frequency band (Fig. 1G). gLFP power significantly increased with contrast (*P* < 0.001; Table 1), and depended significantly on stimulus type (*P* < 0.001; Table 1). Plaid pattern presentation resulted in significantly lower gLFP power than did grating presentation (*P* < 0.001; Table 1). Figures 1E–G show a breakdown of the effects for the different recording sites for three different contrasts (8%, 16%, and 48%) and frequency bands. The results for the sites that were non-optimally stimulated by the single grating are shown in Fig. 2. Figure 2A illustrates the stimulus-induced power as a function of frequency for three different contrasts. The stimulus-induced power in the gamma range increased with contrast, but again, this increase appeared to be much weaker with plaid pattern presentation than with grating presentation. Figure 2B shows the distribution of preferred orientation relative to the grating stimulus orientation (which was horizontal; labelled as 0° in Fig. 2B). Figure 2C shows the normalized population firing rate for the different contrasts and the two stimuli. Figure 2D shows these data for the non-normalized rates. Sites not stimulated optimally by the grating showed significantly higher firing rates when the plaid pattern was presented (*P* < 0.001; for details regarding significance levels, see Table 1). Figure 2C and D shows the results for the individual recording sites and three different contrasts. Most data points fall to the left of the diagonal, showing that plaid pattern presentation resulted in higher firing rates than grating presentation for almost all sites and that the effect was significant (*P* < 0.001, Wilcoxon signed rank test). The corresponding effects on the alpha, beta and gLFP power are shown in Fig. 2E–G. LFP alpha power was significantly affected by contrast and stimulus type (*P* < 0.05). Increasing contrast decreased alpha power, and gratings decreased alpha power, when compared with plaid stimuli (*P* < 0.05). There was no interaction between the two factors (Table 1). Beta power was significantly affected by stimulus type, but not stimulus contrast. There was no interaction between the two factors (Table 1). gLFP power significantly increased with contrast, but this was significantly more prominent when gratings were presented (see Table 1 for details regarding statistical effects). Figure 2E–G shows the effects for individual recording sites for 8%, 16% and 48% contrast stimuli.

**Table 1:** Significance of stimulus manipulations on firing rates and power spectra of selected LFP frequency bands

	Spikes (norm)			Spikes (non-norm)			Alpha power			Beta power			Gamma power		
					
	d.f.	*F*	*P* <(=)	d.f.	*F*	*P* <(=)	d.f.	*F*	*P* <(=)	d.f.	*F*	*P*	d.f.	*F*	*P* <(=)
Pool 1															
Stimulus effect	1	6.5	0.011	1	1.2	0.284	1	14.4	0.001	1	0.3	0.556	1	50.5	0.001
Contrast effect	7	14.5	0.001	7	1.9	0.068	7	14.5	0.001	7	1.7	0.101	7	11.8	0.001
Stim.* contrast	7	0.6	0.73	7	0.1	1.0	7	0.6	0.74	7	0.7	0.644	7	4.2	0.001
Pool 2															
Stimulus effect	1	66.7	0.001	1	11.9	0.001	1	33.5	0.001	1	6.8	0.010	1	65.2	0.001
Contrast effect	7	16.9	0.001	7	1.9	0.073	7	2.4	0.021	7	1.8	0.090	7	7.0	0.001
Stim.* contrast	7	4.0	0.001	7	0.6	0.782	7	1.4	0.224	7	1.1	0.389	7	30.6	0.001
Pool 1 + 2															
Stimulus effect	1	22.5	0.001	1	4.0	0.048	1	54.6	0.001	1	6.3	0.012	1	118	0.001
Contrast effect	7	37.9	0.001	7	5.4	0.001	7	5.9	0.001	7	3.1	0.003	7	11.0	0.001
Stim.* contrast	7	4.6	0.001	7	0.7	0.681	7	1.2	0.274	7	1.4	0.197	7	40.2	0.001
Pool 3															
Stimulus effect	1	18.9	0.001	1	1.17	0.280	1	173.3	0.001	1	569.2	0.001	1	440	0.001
Ori. effect	8	21.9	0.001	8	6.13	0.001	8	1.27	0.25	8	2.15	0.029	8	2.29	0.02
Contrast effect	2	42.8	0.001	2	7.79	0.001	2	121.2	0.001	2	164.9	0.001	2	82.8	0.001
Stim*Ori.	2	12.5	0.001	2	3.5	0.001	2	1.22	0.28	2	0.56	0.81	2	1.63	0.11
Cont.*Ori.	8	0.25	0.99	8	0.03	1	8	0.55	0.91	8	0.36	0.98	8	0.38	0.98
Stim. * Cont.	16	1.05	0.35	16	0.23	0.79	16	16.8	0.001	16	17.2	0.001	16	6.71	0.001
Triple interact.	16	0.1	1	16	0.01	1	16	0.65	0.85	16	0.51	0.94	16	0.3	0.99

Stim., effect of stimulus type; Ori., effect of stimulus orientation; Cont., effect of stimulus contrast; Interact., interaction between main effects. Spikes (norm) refers to the effects on normalized firing rates, and Spikes (non-norm) refers to the effects on non-normalized firing rates. Alpha power refers to the effects on the averaged power in a frequency band ranging from 7 to 13 Hz, beta power to the effects on the averaged power in a frequency band ranging from 13 to 25 Hz, and gamma power refers to the effects on the averaged power in a frequency band ranging from 30 to 60 Hz. *P*-values were derived by employing an anova [d.f., degrees of freedom; *P* <(=), *P*-value of the factor of interest].

* Interaction between factors of interest. For description of pool 1, pool 2, pool 1 + 2, and pool 3, see main text.

**Fig. 3:**
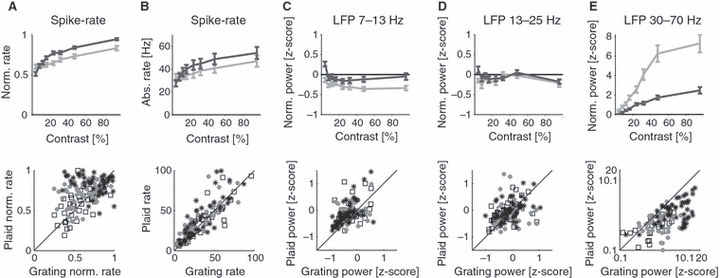
Comparison of LFP power and multi-unit spiking activity for all sites irrespective of their preferred orientation relative to the orientation of the grating stimulus. (A) Top: average normalized spiking activity for different contrasts when gratings (black) and plaid pattern (grey) were presented. Bottom: data from individual recordings (stars, 48%; grey dots, 24%; open squares, 8% contrast). (B) Top: average non-normalized spiking activity for different contrasts when gratings (black) and plaid pattern (grey) were presented. Bottom: data from individual recordings (stars, 48%; grey dots, 24%; open squares, 8% contrast). (C) Top: Average LFP alpha power for different contrasts when gratings (black) and plaid pattern (grey) were presented. Bottom: data from individual recordings (stars, 48%; grey dots, 24%; open squares, 8% contrast). (D) Top: average LFP beta power for different contrasts when gratings (black) and plaid pattern (grey) were presented. Bottom: data from individual recordings (stars, 48%; grey dots, 24%; open squares, 8% contrast). (E) Top: average gLFP power for different contrasts when gratings (black) and plaid pattern (grey) were presented. Bottom: data from individual recordings (stars, 48%; grey dots, 24%; open squares, 8% contrast). Error bars denote SEMs.

Thus, these data suggest that plaid pattern presentation should result in increased firing rates, when averaged across all sites, and it should result in reduced gLFP power as compared with grating presentation. The data averaged across all recording sites are in line with this suggestion (Fig. 3). Figure 3A and B shows the spiking activity (normalized and non-normalized, respectively) as a function of contrast and stimulus type. Contrast and stimulus type both had a significant effect on firing rate (Table 1). Importantly, plaid pattern presentation significantly increased V1 firing rates. Alpha power was significantly reduced as contrast increased (Table 1), and stimulus type also had a significant effect on alpha power (stronger reduction with gratings than with plaid pattern; Table 1). Beta power was affected in a similar manner (the effects were significant, but visual inspection of Fig. 3 suggests that they were somewhat smaller than those for alpha power; Table 1). The largest changes with increasing contrast and altering stimulus type occurred in the gamma frequency range. Contrast significantly increased gLFP power (Table 1), and gratings resulted in significantly larger gLFP power than plaid stimuli (Table 1). Thus, increasing luminance contrast increases average multi-unit activity and gLFP power irrespective of stimulus type, while altering the stimulus type from grating to plaid pattern significantly increased the firing rate at our recording sites, and simultaneously decreased the oscillatory LFP activity in the gamma range.

As seen in Fig. 1, the firing rate for the grating was slightly higher than the firing rates for the plaid pattern when we averaged across sites that were stimulated with a grating orientation within 30° of the preferred orientation. This is evidence for cross-orientation inhibition. Cross-orientation inhibition might have even been more profound had we used stimuli that matched the neuron's preferred orientation more closely. Theoretically, this could result in effects profound enough to cancel or at least reduce the effects shown in Fig. 3A and B, where, on average, plaid pattern increased firing rates. Although we believed this to be unlikely, we investigated this possibility further. Random sampling of neurons with varying preferred orientations, with the stimulus orientation fixed, is not an efficient approach with which to investigate the above possibility. A more economical and systematic approach is to record the neuronal activity when the grating (and plaid pattern) orientation is parametrically altered in small steps, such that some are very close to the peak of the neuron's orientation tuning curve, and others are progressively more distant. We thus recorded the spiking and LFP activity at a total of 14 additional sites while we presented gratings of nine different orientations (0°, 20°, 40°, 60°, 80°, 100°, 120°, 140°, and 160°) and three different contrasts (16%, 32%, and 64%), and while we presented the corresponding plaid pattern. The spiking activity at these sites showed pronounced orientation tuning (showing an orientation index of > 0.5 at each site) when confronted with a grating of 64% contrast. The neurons from this sample are referred to as pool 3 in Table 1.

**Fig. 4:**
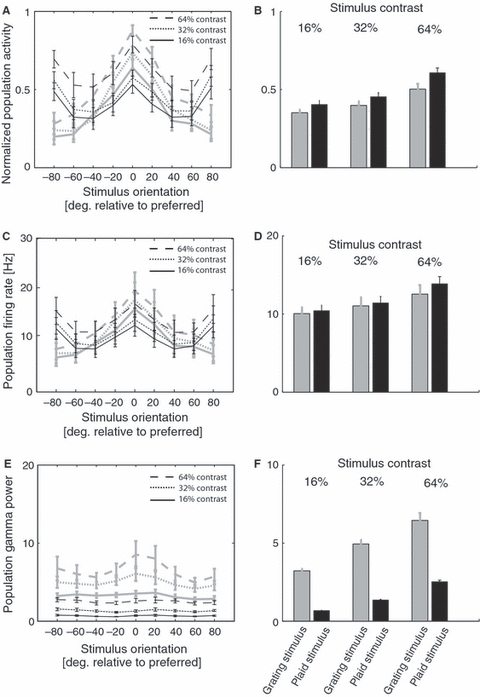
Comparison of gLFP power and multi-unit spiking activity as a function of stimulus orientation and contrast. (A) Normalized population activity from 14 recording sites aligned with the preferred orientation of each site (labelled as 0°). Grating orientation tuning curves are shown in grey, and plaid pattern tuning curves are shown in black. (B) Average response across all different stimulus orientations for the three contrasts and two stimulus types (grey, gratings; black, plaid pattern). (C) Same as A, but for non-normalized spiking activity. (D) Same as B, but for non-normalized spiking activity. (E) Same as A, but for stimulus-induced gLFP power. (F) Same as B, but for stimulus-induced gLFP power. Error bars denote SEMs.

The results of this manipulation are shown in Fig. 4. Figure 4A shows the normalized population tuning curve (aligned to the preferred orientation of each site as assessed with gratings) for the different stimulus contrasts and the different stimulus types (grating vs. plaid). When gratings were presented, the population tuning curves showed a peak at the preferred orientation, and a significant reduction as the difference between preferred orientation and presented orientation increased (*P* < 0.001, three-factor anova, main effect orientation; Table 1). The overall activity levels reduced with contrast (*P* < 0.001, three-factor anova, main effect contrast). When plaid patterns were presented, we found that overall firing rates were lower when one of the component gratings was of the preferred orientation and the other was orthogonal to the preferred orientation, indicating cross-orientation inhibition. Investigation of this for the data obtained at the preferred orientation (0° in Fig. 4) showed that the reduction was significant for the 32% contrast stimuli (*P* < 0.05, signed rank test), but not for the 64% (*P* = 0.497, signed rank test) or 16% (*P* = 0.23, signed rank test; see also Supporting Information) contrast stimuli. As the plaid pattern changed orientation, we found an initial reduction as the difference between preferred orientation and presented plaid orientation increased. This reduction was largest at ∼40° difference (as now both component gratings constituting the plaid were tilted away from the preferred orientation of the neuron). Thereafter, the activity levels increased again, as now the component grating of the plaid that was originally orthogonal to the preferred orientation approached the preferred orientation. There was a significant main effect of stimulus type on overall normalized activity levels (grating vs. plaid pattern, *P* < 0.001, three-factor anova; Table 1). Figure 4B shows the averaged activity across all stimulus orientations for the grating and the plaid pattern. The plaid pattern induced higher average firing rates, and the difference was significant for all three contrast levels (*P* < 0.001, signed rank test). Figure 4C and D shows the respective data for the non-normalized firing rates. Significance values relating to Fig. 4C are listed in Table 1. The non-normalized firing rates showed no significant main effect of stimulus type, but a significant interaction between stimulus type and orientation. This was attributable to slightly higher firing rates for gratings of the preferred orientation, but substantially lower firing rates for gratings of orientations close to the non-preferred orientation, when compared with the plaid pattern. Averaging across all stimulus orientations (Fig. 4D) yielded significantly higher firing rates for the plaid pattern for all stimulus contrasts (*P* < 0.01, signed rank test). Figure 4E and F show the respective data for the gLFP power. gLFP power showed some orientation tuning for gratings of 32% and 64% contrast, but not for lower contrasts or for plaid pattern of any contrast. Plaid pattern resulted in significantly reduced gLFP power (see Table 1 for details regarding the anova statistics). Averaging across orientations (Fig. 4F) yielded significantly higher gLFP power for gratings than for plaid pattern across all contrasts (*P* < 0.001, signed rank test). For a corresponding analysis regarding the alpha and beta power, see Table 1 and Supporting Information. A more detailed analysis regarding the effects of stimulus orientation and contrast on cross-orientation inhibition and the possible effects on BOLD fMRI is provided in the Supporting Information.

To summarize, we found that plaid pattern resulted in significantly higher neuronal spiking activity, but significantly reduced gLFP power activity. This finding replicates the finding from our approach where we randomly sampled from sites that had different preferred orientations, but where we kept the stimulus orientation fixed (Figs 1–3). Having established a robust and consistent stimulus-induced dissociation between spiking activity and gLFP power at the population level, we employed identical stimulus conditions in fMRI experiments, where monkeys passively fixated during stimulus presentation, to determine whether fMRI BOLD signals would follow spiking activity or the changes in gLFP power.

### fMRI results

We used a block design to investigate the influence of grating and plaid pattern presentation on fMRI BOLD responses in two awake fixating monkeys. Stimuli were presented for 15 s, and this was followed by an 18-s blank period. Animals were required to fixate throughout the 33 s (see Materials and methods), and received a generous liquid reward thereafter. Only trials where animals fixated throughout the block were included for further analysis. In total, we obtained 10 data sets from monkey C (three at 48% and seven at 96% contrast) and 46 data sets from monkey W (two at 8%, six at 12%, nine at 16%, four at 24%, five at 48% and eight at 96% contrast with a stimulus duration of 15 s, and another 12 at 96% contrast with a stimulus duration of 3 s). Although our stimuli resulted in robust BOLD signal changes in a variety of visual areas, we restricted our analysis to V1, as we aimed to compare BOLD responses with our electrophysiological V1 recordings. Figure 5A shows voxels with significant activation in V1 co-registered with a structural image from one experimental session. We analysed the BOLD signal change as a function of stimulus type following stimulus onset within our ROI. The ROI consisted of those voxels that were commonly activated between the two conditions and had a z-threshold > 3 (cluster threshold of *P* < 0.05). Table 2 gives an overview of the average ROI size identified for the different experiments and the different coils (and imaging parameters) used. Supporting Information Figure S1 shows the aggregate ROI size and locations that were obtained with different coils. Although we tested other approaches to define our ROI, this criterion allowed us to identify those data sets where activation was present in both conditions, and to discard others where activation was not present or present only in one condition. The inclusion of those data sets would only increase the variance of the BOLD signal change. However, it is important to note that, even when using an ROI that included all activated voxels (irrespective of the stimulus type that elicited them), we obtained similar results (see Materials and methods). Figure 5B shows an example of the percentage signal change for the grating and the plaid pattern presented at 96% contrast. Presentation of the grating resulted in a significantly larger BOLD signal change than presentation of the plaid pattern within the V1 ROI. Figure 5C shows the percentage BOLD signal change for the two stimulus types at 96% contrast averaged across eight experimental sessions for monkey W. Both monkeys showed a significant interaction between stimulus type (plaid pattern/grating) and time (two-way repeated measures anova: monkey W, *F*_10,70_ = 2.413, *P* < 0.05; monkey C, *F*_10,60_ = 2.669, *P* < 0.05). This basic pattern was observed across all stimulus contrasts, as shown in Fig. 6. Figure 6A shows that increasing stimulus contrast resulted in increased percentage BOLD signal change for grating and plaid pattern (*F*_5,28_ = 13.614, *P* < 0.001, main effect of contrast, two-way mixed design anova). However, for all stimulus contrasts, the average percentage BOLD signal change amplitude was significantly lower for plaid pattern than for gratings (*F*_1,28_ = 12.248, *P* = 0.0016, main effect of stimulus type, two-way mixed design anova). A breakdown of effects for the different experimental sessions in monkey W is shown in Fig. 6B. Data acquired with different stimulus contrast are color coded. A Wilcoxon signed rank test showed that the percentage BOLD signal change induced by gratings was significantly larger than the percentage BOLD signal change induced by plaid pattern (*P* = 0.0021).

**Table 2:** Average size of the selected ROI and the respective number of experiments as a function of stimulus contrast for the two animals

	Contrast (%)	No. of experiments	Mean no. of voxels ± SEM
Monkey W	8	2	12 ± 8
	12	6	73 ± 23
	16	9	82 ± 13
	24	4	45 ± 17
	48	5	62 ± 38
	96	20	34 ± 5
Monkey C	48	3	3 ± 1
	96	7	7 ± 3

SEM, standard error of the mean. Note that activation levels in monkey C were generally lower and data were more noisy. Both factors resulted in the reduced ROI sizes for this monkey.

Stimulus duration differed between the electrophysiological data and the BOLD fMRI data, which could have resulted in the stimulus-induced response pattern seen. To control for this possibility, we recorded data from an additional 12 sessions (96% contrast) where the stimulus duration was 3 s, thus matching the electrophysiology stimulus duration. The resulting percentage fMRI BOLD signal change for the two stimulus types in these sessions is shown in Fig. 6B. These data show that plaid pattern presented for 3 s also resulted in lower fMRI BOLD signal change than gratings, and that the effect was significant on its own (*P* < 0.01, signed rank test).

**Fig. 7:**
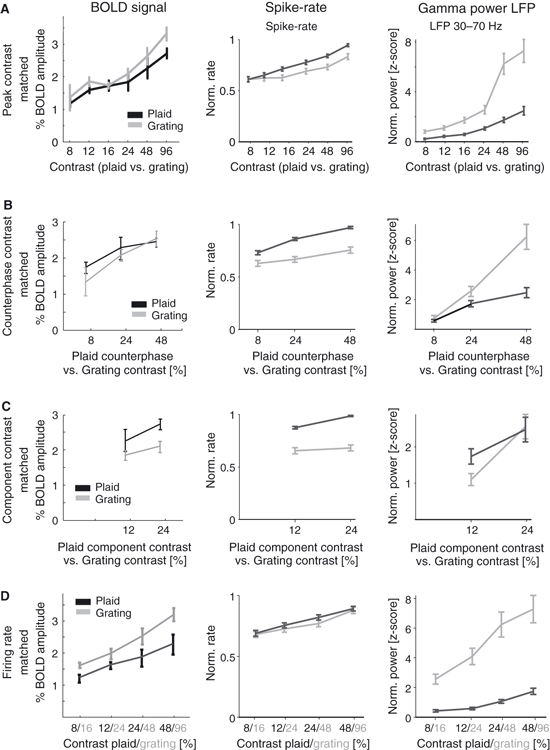
fMRI BOLD, spiking activity and normalized gLFP comparisons re-plotted for different grating/plaid pattern contrasts. (A) Data plotted in a peak contrast-matched manner (i.e. peak contrast of the plaid pattern matches the contrast of the grating). These data are thus identical to the plots shown in Figs 3 and 6A, other than the scaling of the *x*-axis. (B) Comparison of responses when the grating had a contrast that was half the peak contrast of the plaid pattern. (C) Comparison of responses when gratings had one-quarter of the peak plaid pattern contrast, i.e. a component contrast-matched condition (note that the counterphase plaid pattern is effectively composed of four gratings of one-quarter the plaid pattern contrast). (D) Comparison of gratings with twice the plaid pattern peak contrast, which is effectively a comparison of plaid pattern and gratings that generated similar firing rates.

The comparison made so far has been between data for gratings and plaid pattern that were matched in terms of peak contrast. As plaid pattern are composed of multiple ‘component’ gratings, the ‘appropriate’ comparison might be between matched component contrasts. We put the term ‘appropriate’ in apostrophes because it is questionable whether an ‘appropriate’ comparison exists. Nevertheless, we compared responses to gratings and plaids where plaid pattern peak contrasts were multiples of the grating contrast (multiples of 4, 2, and 0.5). These data are shown in Fig. 7. In Fig. 7A, the originally presented data are re-plotted to aid comparison. Figure 7B shows the data obtained when the plaid pattern peak contrast was twice the grating contrast. This would normally be the ‘appropriate’ comparison if simple plaid pattern had been used (a plaid pattern composed of two gratings with 24% contrast would have a peak contrast of 48%). As we used counterphase plaid pattern, the comparison in Fig. 7B is effectively a comparison of ‘orientation’-matched component contrast (a counterphase grating is composed of two gratings that have opposite directions of motion, but identical orientations). These two stimuli yielded no significant difference in fMRI BOLD signal (*P* > 0.05), but a significant increase in spiking activity (*P* < 0.001), and a significant decrease in gLFP power for the plaid pattern (*P* < 0.001). These stimuli thus yielded a dissociation of firing rates and gLFP power from fMRI BOLD signals. Figure 7C shows data for the component contrast matching conditions, comparing the responses elicited by a 24% contrast grating and a 96% peak contrast plaid pattern (12% grating and 48% plaid pattern, respectively). This comparison yielded significantly increased fMRI BOLD activity (*P* < 0.05) and significantly increased spiking activity (*P* < 0.001) for the plaid pattern, but no significant differences in gLFP power (main effect of stimulus, *P* > 0.05; stimulus × contrast interaction, *P* > 0.05). Finally, we compared fMRI BOLD and gLFP power for stimuli that yielded identical firing rates. If fMRI BOLD was an unequivocal predictor of firing rate, changes in the fMRI BOLD activity should be identical for the two stimuli. The data are presented in Fig. 7D. Here, a 48% plaid pattern was compared with a 96% grating, a 24% plaid pattern with a 48% grating, etc. Firing rates under these two stimulus conditions did not differ significantly (main effect of stimulus, *P* > 0.05; stimulus × contrast interaction, *P* > 0.05). However, fMRI BOLD activation and gLFP power were significantly larger for gratings (*P* < 0.001). The latter comparison shows that large BOLD differences can occur in the absence of firing rate differences. To conclude, the BOLD signal change in V1 of the awake macaque monkey generally follows changes in gLFP, and not spiking activity, when these are dissociated or anti-correlated.

## Discussion

We used a simple stimulus manipulation that resulted in a dissociation of population spiking activity and gLFP power to investigate the neuronal basis of the fMRI BOLD signal. Plaid pattern resulted in higher V1 population firing rates than gratings, while at the same time resulting in substantially reduced gLFP power. The fMRI BOLD signal changes were associated with changes in gLFP power, not with changes in spiking activity.

A variety of studies have found that gLFP power is more strongly coupled to neurometabolic activity (and thus the fMRI BOLD signal) than is spiking activity (Logothetis *et al.*, 2001; Niessing *et al.*, 2005; Viswanathan & Freeman, 2007; Goense & Logothetis, 2008; Lippert *et al.*, 2010). However, in many of these studies, gLFP power and spiking activity were correlated (Logothetis *et al.*, 2001; Niessing *et al.*, 2005; Goense & Logothetis, 2008). Furthermore, Burns *et al.* (2010) argued that multi unit activity is as sustained as LFP, and that therefore these two extracellularly recorded signals are as likely to be correlated with the BOLD signal. A few studies have shown that gLFP power and spiking activity can be de-correlated or anti-correlated. Viswanathan & Freeman (2007) have shown that thalamocortical inputs to V1 follow high temporal frequency stimulation (32 Hz), and thus affect gLFP power, without being able to drive spiking activity in V1 of anaesthetized cats. Lippert *et al.* (2010) have reported that opponent motion stimuli can result in reduced firing rates in some recordings of middle temporal area of the macaque (sites that showed little direction selectivity), whereas they may increase gLFP power and the simultaneously acquired local fMRI BOLD signal at those sites. Both studies are in line with our finding from V1, namely a de-correlation of spiking and gLFP power, where the fMRI BOLD signal (or the neurometabolic signal) follows changes in gLFP power. We also show that such decoupling does not necessarily require artificial stimulus conditions, but might occur regularly in large parts of V1 under natural viewing conditions. Whereas gratings and plaid patterns are both artificial stimuli, most natural images contain a multitude of orientations, which often overlap, rather than just a single extended orientation on a grey background. Thus, the plaid pattern may be closer to a natural image than a single grating, however artificial both really are. The finding of reduced gLFP power with plaid pattern presentation also suggests that natural images might not elicit gLFP power of notable size in V1, although, to the best of our knowledge, this has not been parametrically studied. These considerations highlight how important it is to understand the effect of stimulus manipulations on the local neuronal circuitry. Without such an understanding, inference of spiking activity from the BOLD signal may be seriously misguided. Equally, it is essential to understand how task or perceptual manipulations affect the local neuronal circuitry for adequate interpretation of the fMRI BOLD signal. This has been demonstrated for perceptual flash suppression in V1, where BOLD signal changes occurred in the near absence of changes in V1 firing rates, although low-frequency LFP fluctuations, rather than gLFP oscillations, were related to the BOLD changes (Maier *et al.*, 2008). Recent data from V1 demonstrate that very focal attention to the centre of the RFs reduces gamma frequency oscillations, while simultaneously increasing neuronal firing rates (Chalk *et al.*, 2010). At least the voxels representing the focus of attention should therefore show a reduction in BOLD activity, according to our current data. However, attention invariably increases BOLD activity in V1 in human fMRI (Huk & Heeger, 2000; Jack *et al.*, 2006; Smith *et al.*, 2006; Silver *et al.*, 2007; Murray, 2008), whereas BOLD responses to unattended stimuli are suppressed in V1 (Slotnick *et al.*, 2003). Whether an attention-induced BOLD increase is attributable to increased firing rates in the presence of reduced gamma oscillations, or whether different mechanisms are in play, remains to be determined.

Is there a common denominator that explains the discrepancy between neurometabolic and spiking activity changes in our and other published studies? Such a common denominator may be how strongly the inhibitory network is modulated, but even this idea is somewhat tenuous. The inhibitory local network is strongly activated if stimuli are presented that extend beyond the classic RF (Sengpiel *et al.*, 1997; Walker *et al.*, 2000; Jones *et al.*, 2001), which would have been the case for our 3° stimuli. This suppression is maximal when the centre and surround are stimulated with the same orientation and spatial frequency (Knierim & van Essen, 1992; DeAngelis *et al.*, 1994; Angelucci *et al.*, 2002). Another source of inhibition could arise from within the classic RF. Cross-orientation inhibition (DeAngelis *et al.*, 1992; Carandini *et al.*, 1997; Sengpiel *et al.*, 1998) [also called masking (Koelling *et al.*, 2008)] is mostly confined to the classic RF itself (DeAngelis *et al.*, 1992). Despite apparent suppression of responses by the mask, the extent to which masking involves the intracortical inhibitory network is debated (Freeman *et al.*, 2002; Koelling *et al.*, 2008). Retinal contrast gain control (Shapley & Victor, 1981), suppression in the lateral geniculate nucleus (Freeman *et al.*, 2002) and synaptic depression at the thalamocortical synapse (Freeman *et al.*, 2002) could be sufficient to explain cross-orientation inhibition (masking). There nevertheless appears to be good evidence for a role of inhibition in cortical information processing beyond surround suppression/contrast normalization (Anderson *et al.*, 2000; Monier *et al.*, 2003; Shapley *et al.*, 2003; Xing *et al.*, 2005).

How could inhibition affect spiking and gLFP power? In the study of Lippert *et al.* (2010), motion opponency would increase the inhibitory drive, resulting in increased gamma oscillations and a reduced firing rate (Traub *et al.*, 1996; Whittington & Traub, 2003; Gieselmann & Thiele, 2008). However, a plaid pattern should invoke cross-orientation inhibitory mechanisms (Nelson & Frost, 1978; Sengpiel *et al.*, 1998) and thus also increase gamma frequency oscillation, but the opposite was found in our study. It could be argued that the reduced gLFP power with plaid pattern presentation was attributable to reduced contrast normalization, as plaid pattern components were effectively one-quarter of the grating contrast. Indeed, when plaid pattern components were matched to the grating contrast (Fig. 7C), gLFP power did not differ significantly between the two conditions. Although this is an intriguing possibility, it does not withstand scrutiny. Lima *et al.* (2009) reported reduced gLFP power in V1 when (square wave) plaid pattern were composed of components that matched the grating contrast. Thus, the reduced gLFP power seen upon plaid pattern presentation is not solely a result of reduced contrast normalization. However, masking as a result of depression at the thalamocortical synapse (Freeman *et al.*, 2002) could partly explain these findings. Depression at the thalamocortical synapse might reduce cortical excitation and inhibition, resulting in lower gLFP power.

If, as suggested above, inhibitory drive was the main determinant of gLFP power, how would this relate to BOLD fMRI? It is often argued that inhibitory synaptic activity is less metabolically demanding than excitatory synaptic activity (Logothetis, 2002). Why would increased gamma frequency oscillations increase the fMRI BOLD signal? If the increased inhibitory activity was driven by pyramidal – inhibitory neuronal interactions (Tiesinga & Sejnowski, 2009), then the overall synaptic activity in the network might increase, even if cells were only permitted to spike during a short phase of the gamma cycle. However, as the large majority of synaptic connections within an area originate from within the area itself (Douglas *et al.*, 1995), it is not straightforward to envisage strong, continued synaptic activity during periods of reduced spiking activity. Therefore, it is also difficult to explain increased neurometabolic demand in the presence of reduced excitatory synaptic activity, if the latter is the main determinant of metabolic demand.

Irrespective of these unresolved questions, our data show that it can be difficult to predict *a priori* what result a specific stimulus (or task) manipulation has on the local network. It is thus equally difficult to predict whether gLFP power and spikes increase or decrease together, or whether they become decoupled. Given this, it is also difficult to interpret the fMRI BOLD response adequately without *a priori* knowledge of the underlying electrophysiology.

## Abbreviations

BOLD: blood oxygenation level-dependent
BPS: baseline power spectrum
BPS_M_: mean of the baseline power spectrum
BPS_SD_: standard deviation of the baseline power spectrum
EPI: echo-planar imaging
fMRI: functional magnetic resonance imaging
FOV: field of view
gLFP: local field potential in the gamma range
LFP: local field potential
MDEFT: modified driven equilibrium Fourier transform
MRI: magnetic resonance imaging
PSz: stimulus-induced power spectrum
RF: receptive field
ROI: region of interest
SPS_M_: mean stimulus power spectrum
TE: echo time
TR: repetition time
V1: primary visual cortex

## Supporting Information

**Fig. S1.** ROIs used in monkey C to extract %BOLD signal.

**Fig. S2.** Cross orientation inhibition at the preferred grating orientation for 3 different stimulus contrasts.

**Fig. S3.** Influence of stimulus orientation, contrast and type on LFP alpha and beta power.

Please note: As a service to our authors and readers, this journal provides supporting information supplied by the authors. Such materials are peer-reviewed and may be re-organized for online delivery, but are not copy-edited or typeset by Wiley-Blackwell. Technical support issues arising from supporting information (other than missing files) should be addressed to the authors.
