# Supplementary material for: Stimulus-induced dissociation of neuronal firing rates and local field potential gamma power and its relationship to the blood oxygen level-dependent signal in macaque primary visual cortex
Source: Eur J Neurosci. 2011 Dec;34(11):1857–70. doi: 10.1111/j.1460-9568.2011.07877.x (PMC3274700; doi:10.1111/j.1460-9568.2011.07877.x)
Supplement: Supplementary file 1 [file ejn0034-1857-SD1.doc]

**Supporting Information**

**Stimulus induced dissociation of neuronal firing rates and LFP gamma power and its relation to the fMRI BOLD signal in macaque V1**

M.J. Bartolo^1^, M. A. Gieselmann^1^, V. Vuksanovic^1^, D. Hunter^1^, L. Sun^1^, X. Chen^1^, L.S. Delicato^2^, A. Thiele^1*^

^1^Institute of Neuroscience, Newcastle University, Newcastle upon Tyne, NE2 4HH, UK.

^2^Current address: Department of Psychology, School of Business, Law & Psychology, University of Sunderland, Sunderland, SR6 0DD,

*corresponding author: A. Thiele, Institute of Neuroscience, Newcastle University, Newcastle upon Tyne, NE2 4HH, UK, e-mail: [alex.thiele@ncl.ac.uk](mailto:alex.thiele@ncl.ac.uk)

**Effect of coils, scanning parameters, and stimulus presentation device on locations and sizes of fMRI regions of interest (ROI)**

As described in the main paper we used 2 different surface coils for the fMRI data acquisition, somewhat different RF pulse parameters, and also different visual presentation systems over the course of the experiments (which were acquired over a total of 5 years, although the majority was acquired within the last 1.5 years). Figure 1 shows the aggregate ROI location projected onto the axial plane of a co-registered MDEFT scan. To generate the aggregate ROIs we first co-registered all the different EPI data sets to a single EPI data set. We then co-registered that single EPI data set to a structural data set (MDEFT) that was acquired in the same session, and superimposed the aggregate ROI onto the structural scan.


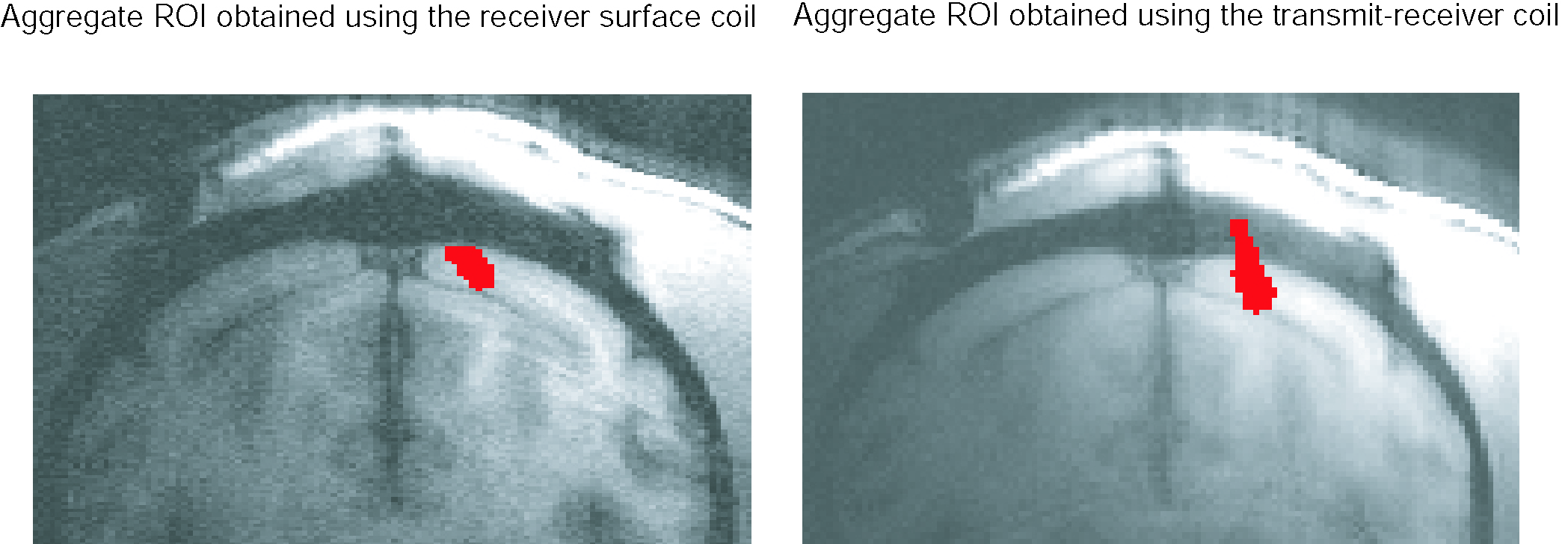


Supporting Information Figure S1: ROIs used in monkey C to extract %BOLD signal. Data are from two different coils. The ROIs are located in similar locations medio-laterally. The anterior-posterior variance of the ROI is due to the difficulty of adequate co-registration between EPIs and then finally to an MDEFT (structural) scans. This difficulty arises because only small (and sometimes varying) parts of the brain show high image contrast (a consequence of the surface coil use).

Supporting Information Figure S1 shows that the different coils resulted in similar ROI locations, at least in relation to the medio-lateral location on the operculum. The apparent extension into what would normally considered to be V2 (left and right image) and outside the brain (right image), is due to the difficulty of registering different surface coil EPI data to one another with a final step of registration to a surface coil MDEFT data set. Some misalignment occurs in each EPI to EPI registration step, as the signal drop off can vary between EPI data. These registration difficulties are particularly prominent in the phase encoding direction (anterior-posterior direction).

**Plaid pattern presentation and cross orientation inhibition**

Our data allowed us to assess to what extent plaid pattern stimulus presentation resulted in cross orientation inhibition and/or cross orientation facilitation. Cross orientation inhibition is usually most profound at the preferred orientation of a neuron. Our data presented in figure 1 of the main paper shows evidence of cross orientation inhibition, but it was not very profound. It could be argued that our selection of neurons could have caused this weak cross orientation inhibition, as we selected neurons who’s preferred orientation was within 30 degrees of the grating orientation that was presented. Had we used a stimulus that was closer to the ‘real’ preferred orientation we might have seen more evidence of cross orientation inhibition, and this could have pointed to less of a dissociation between LFP gamma power, spiking, and BOLD activity overall.

In order to properly assess the amount of cross orientation inhibition we measured the activity when at least one of the gratings was very close to the preferred orientation, while the other is 90 deg tilted (for the plaid pattern condition). We recorded the spiking and LFP activity of 14 neurons while presenting gratings of 9 different orientation (0, 20,40,60,80,100,120, 140,160 deg) and 3 different contrasts (16, 32, 64%), and while we presented the corresponding plaid pattern. The spiking activity at these sites showed pronounced orientation tuning (showing an orientation index of >0.5 at each site) when confronted with a single grating of 64% contrast. Figure 4 of the main manuscript shows that cross orientation inhibition was on average present at the preferred orientation, evident by the fact that the responses to gratings of optimal orientation were somewhat higher than the responses to the corresponding plaid pattern. Quantification of this difference, however, showed that it was only significant for the 32% stimulus condition (p=0.0052, paired t-test, see Supporting Information Figure S2), not for the 64% (p=0.4973, paired t-test, see Supporting Information Figure S2), or the 16 % stimulus condition (p=0.2676, paired t-test, see Supporting Information Figure S2). This was the case irrespective of whether we used normalized or non-normalized data sets for testing.


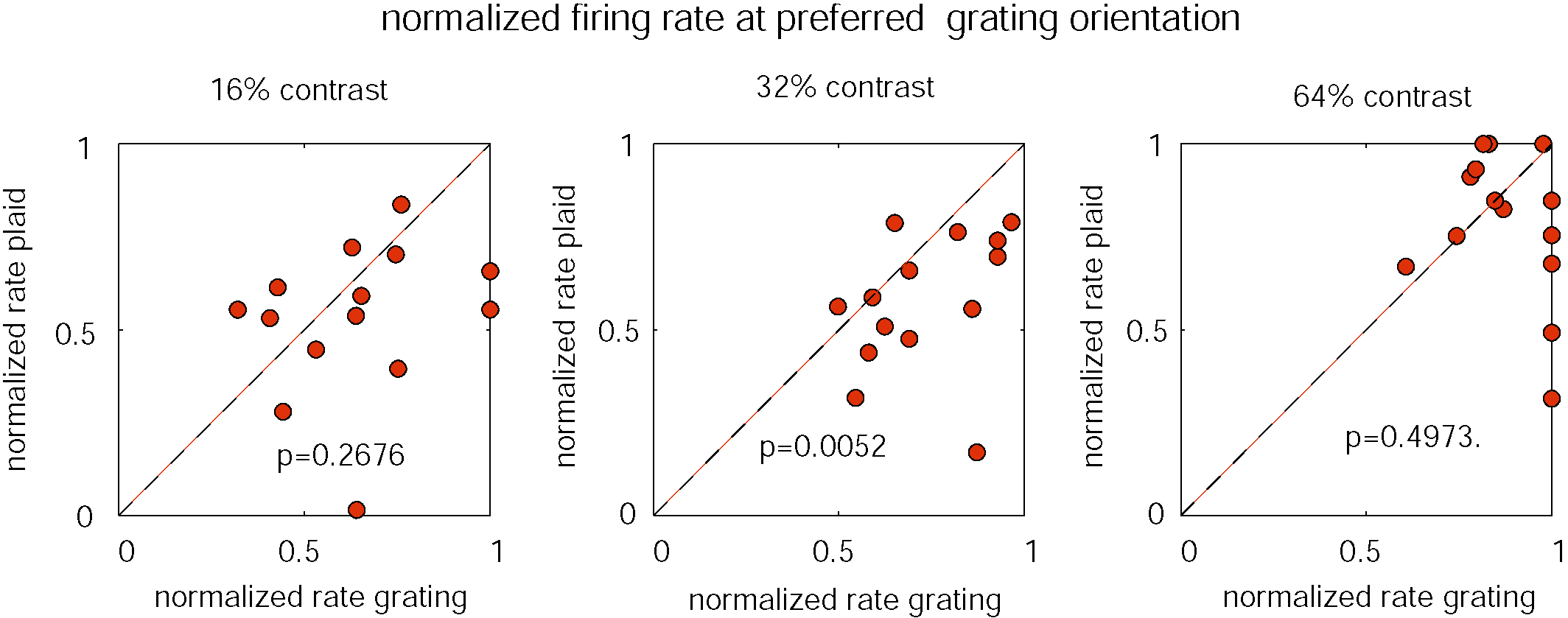


**Supporting Information Figure S2**: Cross orientation inhibition at the preferred grating orientation for 3 different stimulus contrasts. Each data point shows the normalized neuronal activity (normalized to the maximum across all contrasts, stimuli, and orientations) for a cell, when grating stimuli (x-axis) and plaid stimuli (y-axis) were presented. P-values denote the significance of the effect (paired t-test).

Thus, cross orientation was not much more pronounced when cells were stimulated close to the preferred orientation, and hence the dissociation between spikes, gamma band LFP and fMRI BOLD signal was comparable in the two approaches we took (fixing the stimulus orientation with random sampling of neurons with different preferred orientation vs. varying the orientation parametrically for each recorded site). However, even if we had found stronger cross orientation inhibition in the experiment where orientation was varied parametrically, it would not necessarily have invalidated our finding presented in figures 1-3 of the main manuscript. It is important to reiterate that cross-orientation inhibition at the preferred orientation is only a small part of the picture that is relevant for the comparison of spiking activity and fMRI BOLD activity. If spikes were the driving force behind BOLD changes, then the total number of spikes that occur in any given voxel would determine the BOLD signal amplitude, not just the spikes of neurons that are stimulated with the preferred orientation (and the cross orientation, in the case of cross orientation inhibition). The voxels in our study (and most fMRI studies) are much larger than a single orientation column in V1, and thus it is important to determine the number of spikes across orientation columns, i.e. those that are stimulated optimally, those that are stimulated sub-optimally, and those that are stimulated with the ‘anti’ preferred orientation. These averaged (or summed) spike (activity) numbers then need to be compared to the averaged (or summed) activity numbers that are obtained when the corresponding plaid pattern is presented. Figures 4b, d of the main manuscript shows that these averaged activities were significantly larger for plaid pattern than for grating stimuli, irrespective of stimulus contrast (16%: p=0.0001; 32%: p=0.0071; 64%: p=0.0034, signed rank test). Thus, across hypercolumns of V1, plaid pattern presentation results in stronger overall spiking activity, when compared to the presentation single gratings, and if the number of spikes determined the BOLD signal strength, then plaid pattern should result in larger BOLD activity.

**Cross orientation facilitation?**

Our data in the main manuscript show stronger dissociation between spiking activity and LFP gamma power for non-optimal stimulus orientations. At first glance this might hint at cross orientation facilitation for non-optimal stimulus conditions. However, we are hesitant to interpret the data this way. It is simply the case that presentation of a non-optimally orientated stimulus to an orientation tuned cells resulted in low firing rates. Addition of an optimally oriented stimulus to the non-optimally oriented stimulus increased the input drive, and the neuron fired more. The apparent cross orientation facilitation under these conditions is just a consequence of adding a preferred stimulus to a non-preferred stimulus.

At the same time, Supporting Information Figure S2 suggests that some cells fired more when a non-preferred stimulus was added to a preferred stimulus, which does hint at incidences of ‘true’ cross orientation facilitation. We did not quantify this in detail as it is not of key importance for this paper.

**Influence of different stimulus orientations on alpha and beta stimulus induced LFP power**

In the main manuscript we described the influence of stimulus orientation and contrast on spiking activity and on the LFP gamma power (figure 4 main manuscript). Here we also describe the effect of these manipulations on the alpha and beta power. The data are shown in Supporting Information Figure S3. In the alpha band (Supporting Information Figure S3A) we found no significant main effect of stimulus orientation (p=0.256, 3 factor ANOVA), but stimulus type (p<0.0001, 3 factor ANOVA) and contrast (p<0.0001, 3 factor ANOVA), both had significant main effects. Moreover, we also found a significant interaction between stimulus type and contrast (p<0.0001, 3 factor ANOVA). None of the other interactions were significant. In the beta band (Supporting Information Figure S3B) we found a significant main effect of stimulus orientation (p=0.029, 3 factor ANOVA), of stimulus type (p<0.0001, 3 factor ANOVA) and of contrast (p<0.0001, 3 factor ANOVA). Moreover, we also found a significant interaction between stimulus type and contrast (p<0.0001, 3 factor ANOVA). None of the other interactions were significant.


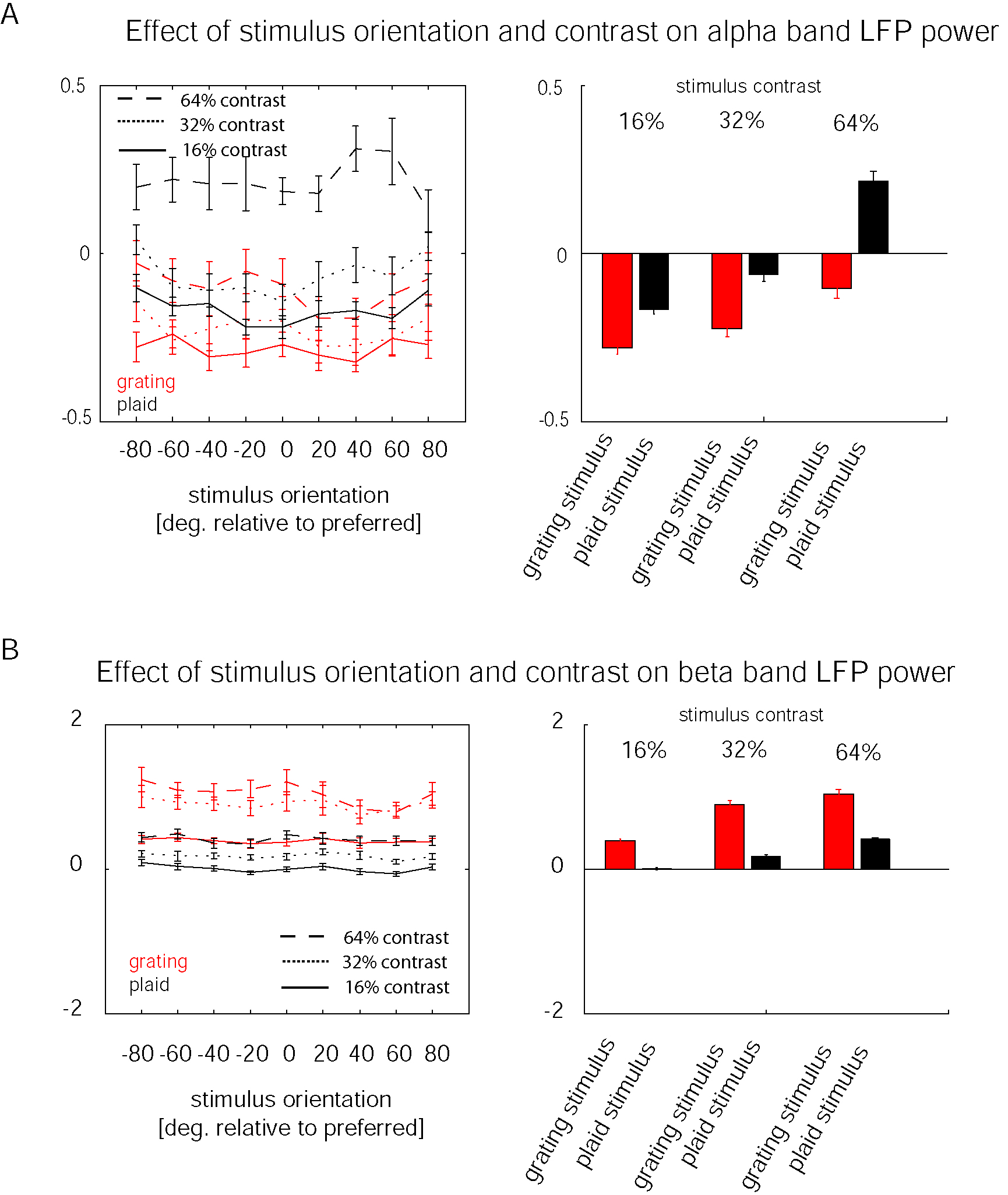


**Supporting Information Figure S3**: Influence of stimulus orientation, contrast and type on LFP alpha and beta power. A) Alpha power was more strongly suppressed for grating than for plaid stimuli. Surprisingly, low contrast stimuli (solid lines) caused the strongest suppression. Alpha LFP power showed no apparent tuning for any of the stimulus types or contrasts. B) Beta LFP power was stronger for grating stimuli, compared to plaid stimuli, and it was stronger for high contrast compared to low contrast stimuli. There could be some indication of orientation tuning (significant main effect of orientation, see text) in the beta frequency band for high contrast gratings, but it is weak at best.

**Details of data fMRI data acquisition**

Supporting Information Tables S1-3 provide a breakdown of the different fMRI experiments performed, which coils were used in any given experiment and what visual display system was used.

Monkey W data acquisition details

| **Contrast** | **TR coil (#runs)** | | **R coil (#runs)** | | **Total** |
| --- | --- | --- | --- | --- | --- |
|  | Res = 0.1*0.125 | Res = 0.1*0.1 | Res = 0.1*0.125 | Res = 0.1*0.1 |  |
| 8 |  |  |  | 2 | 2 |
| 12 |  |  |  | 6 | 6 |
| 16 |  |  |  | 9 | 9 |
| 24 |  |  |  | 4 | 4 |
| 48 | 2 |  |  | 3 | 5 |
| 96 | 7 | 1 |  |  | 8 |
|  | 10 | | 24 | | 34 |

Supporting Information Table S1: Different coils used in monkey W during the different experiments (TR coil:= transmit and receiver coil. R coil:= receiver coil (transmission was done by means of a birdcage coil).

| **Contrast** | **#runs with projector** | **#runs with**  **Avotec** |
| --- | --- | --- |
| 8 | 2 |  |
| 12 | 6 |  |
| 16 | 9 |  |
| 24 | 4 |  |
| 48 | 2 | 3 |
| 96 | 1 | 7 |

Supporting Information Table S2: Different projection systems used in monkey W during the different experiments (projector:= back-projection system . Avotec:= Avotec projection system).

**Monkey C**  data acquisition details

| **Contrast** | **TR coil (#runs)** | | **Bruker coil (#runs)** | | **Total** |
| --- | --- | --- | --- | --- | --- |
|  | Res = 0.1*0.2 | Res = 0.1*0.1 | Res = 0.1*0.2 | Res = 0.1*0.125 |  |
| 48 | 3 |  |  |  | 3 |
| 96 | 4 |  |  | 3 | 7 |
|  | 7 | | 3 | | 10 |

Supporting Information Table S3: Different coils used in monkey W during the different experiments (TR coil:= transmit and receiver coil. R coil:= receiver coil (transmission was done by means of a birdcage coil). All the data were acquired using the back projector system.
